# Knowledge, Attitude, and Practice of Adolescent Girls towards Reducing Malnutrition in Maiduguri Metropolitan Council, Borno State, Nigeria: Cross-Sectional Study

**DOI:** 10.3390/nu12061681

**Published:** 2020-06-04

**Authors:** Ruth Charles Shapu, Suriani Ismail, Norliza Ahmad, Lim Poh Ying, Ibrahim Abubakar Njodi

**Affiliations:** 1 Department of Community Health, Faculty of Medicine and Health Science, University Putra Malaysia, Serdang 43400, Selangor, Malaysia; ruthyshapu52@gmail.com (R.C.S.); lizaahmad@upm.edu.my (N.A.); pohying_my@upm.edu.my (L.P.Y.); 2 College of Nursing and Midwifery, Damboa Road, Maiduguri 600252, Borno State, Nigeria; 3 Department of Physical and Health Education, University of Maiduguri, Maiduguri 600230, Borno State, Nigeria; ibrahimnjodi@gmail.com

**Keywords:** adolescent, malnutrition, knowledge, attitude, practice, information, motivation, behavioral skills

## Abstract

Addressing the gap in knowledge, attitude, and practice among adolescent girls are important as malnutrition has a negative effect on their future generation. This study aimed to determine the knowledge, attitude, and practice of adolescent girls towards reducing malnutrition in Maiduguri Metropolitan Council, Borno State, Nigeria. This was a school-based cross-sectional study conducted among 612 adolescent girls (10 to 19 years old). KoBo collect toolbox was used for the data collection between 3 June and 31 July 2019. Multivariable logistic regression was used to identify predictors of knowledge, attitude, and practice towards reducing malnutrition. The majority of respondents (451, 80.2%; 322, 57.3%) had poor knowledge and attitude towards reducing malnutrition respectively, 278 (49.5%) had poor practice towards reducing malnutrition. Schooling (GGSS; *p* = 0.022; Shehu Garbai; *p* = 0.003) was a significant predictor of knowledge. Religion (*p* = 0.023), information (*p* < 0.001) and motivation (*p* < 0.001) were significant predictors of attitude. School (GGSS; *p* < 0.001; GGC; *p* < 0.001; Shehu garbai; *p* < 0.001; Bulabulin; *p* = 0.030; Zajeri day; *p* = 0.049), education of father (*p* = 0.001), information (*p* = 0.026) and behavioral skill (*p* = 0.019) were significant predictors of practice. There is a need to focus on both school-based and community-based health education intervention to address the poor knowledge, attitude, and practice among adolescent girls for a healthier future.

## 1. Introduction

**Figure 1:**
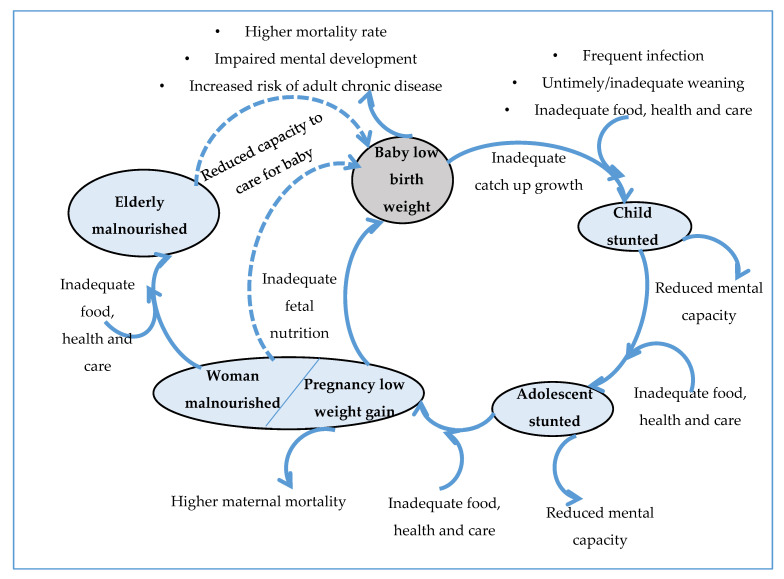
Nutrition throughout the life cycle showing the effect of malnutrition on the adolescent girl and her unborn child [12].

Adolescents are young individuals that are between the ages of 10 and 19 years’ old; this is a period of transition from childhood to adulthood and also a critical phase of physical growth and development [1]. Globally, there are about 1.2 billion adolescents; 90% of them reside in low and middle-income countries, and 125 million lives in areas affected by conflict [1,2]. In Nigeria, adolescents constitute about 23% of the nation’s population [3]. This stage is sensitive to malnutrition as a result of the increased physiological need for nutrition that can be affected by insufficiency, excess, or inequality in individual energy intake, which can affect them and their future generation [1,4,5]. In Nigeria, the prevalence of child marriage before the age of 18 years is 39%, and 16% of adolescent girls are married off before the age of 15 years, resulting to motherhood in childhood. Early marriage exposes adolescent girls to pregnancy complications such cephalon–pelvic disproportion that tends to double the burden of malnutrition, placing the child at higher risk of mortality before their fifth birthday due to little or no information about malnutrition, diet, and nutritional status [6,7,8]. Though malnutrition itself is a problem found among both boys and girls, the adverse effect is more on the girl child. If an adolescent girl enters into the reproductive cycle in a malnourished state, she will grow up into a malnourished adult and give birth to a malnourished child, as shown in Figure 1, contributing to an unproductive community and the cycle of intergenerational transfer of malnutrition. The key to breaking the cycle of intergenerational transmission of malnutrition is to improve the nutrition of adolescent girls, in general, to ensure longer-term sustainable results in reducing malnutrition, poverty, and food insecurity. Without adequate knowledge, attitude, and practice towards reducing malnutrition among adolescent girls and young women before, during and after pregnancy, it will be impossible to have a healthy community [9,10,11,12].

Nigerian demographic and health survey 2013 reveals the prevalence of malnutrition among adolescent girl to be about 23%, similar to a study in Ibadan which showed that 23% of adolescent girls are malnourished [13,14]. Studies among adolescent in private and public schools in Ibadan, Nigeria reveal that 42% of adolescent girls in private schools were underweight, 1.6% were stunted, while 25% of girls in public schools were underweight, and 5.8% were stunted. Studies in port Harcourt reveals that 33.7% of adolescent girls are underweight, and 34.7% are stunted [15,16].

The prevalence of nutritional knowledge was found to be 8.3% in Sokoto state [17]. Inadequate knowledge, attitude, and practice of adolescent girls towards reducing malnutrition place their future and that of the unborn child at risk of pregnancy-related complication, morbidity, mortality, and also the intergenerational cycle of malnutrition and poverty [10,18]. Knowledge itself is a vaccine that empowers the adolescent girl in making choices about preventing malnutrition in the future. There is a link between poor knowledge, motherhood in childhood, and malnutrition, placing some regions at the highest level of malnutrition [7]. Adolescent girls empowered with knowledge, attitude, and practice on malnutrition have about a 40% chance of their children surviving beyond their 5th birthday [19].

**Figure 2:**
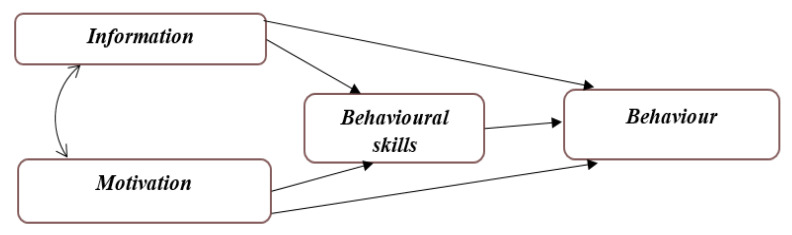
The Information–Motivation–Behavioral Skills Model (IMB). The theory focuses on three components, resulting in a behavioral change. Relevant information leading to the desired behavior, proper motivation to bring about such behaviors, then individuals are equipped with the required skills and abilities to carry out the desired actions.

The use of theory helps in identifying individual features and surrounding environment, to determine characteristics, belief, and personal values associated with various health behavior that is capable of changing the attitude and behavior of an adolescent. Information, motivation, behavioral skill (MB) has three constructs that interact with each other theoretically, resulting from changing in behavior. The Information–Motivation–Behavioral Skills Model (IMB) model has been widely used and has proven to be a useful model in improving healthy practices, as shown in Figure 2, [20,21]. This theory was articulated to malnutrition. The information included the effect and preventive measures of malnutrition. Motivation includes personal motivation and social support from family and friends about eating varieties of food. Behavioral skill identified how hard or easy it is to assess, store, and cook nutritious food. The use of theory will inform adolescents in reducing malnutrition based on the information acquired, their personal view, and the support they get from family and friends. The outcome from the theory aspect will guide future studies in developing the intervention module, especially in terms of skills to overcome the difficulties with regards to assessing, storage, and preparation of food using local means.

There are several gaps in knowledge, attitude, and practice among adolescent girls towards malnutrition, serving as barriers to adolescent health. Many countries and regions have limited information on the health needs, magnitude, and burden of malnutrition among these age groups [19]. This is an opportunity for improving the health and development of these age groups in malnutrition, macronutrient, micronutrient, and healthy eating to significantly improve their nutritional status [22].

Therefore, to be able to overcome the emerging issue of malnutrition among adolescent girls, this study was conducted to determine knowledge, attitude, and practice of adolescent girls towards reducing malnutrition in Maiduguri Metropolitan Council, Borno state, Nigeria using information, motivation, behavioral skill model.

## 2. Materials and Methods

### 2.1. Study Location

The study location was Maiduguri, the Borno state capital, Nigeria, usually called Yerwa by its citizens. It is one of the 27 local government council of Borno state, situated at latitude 11.85, longitude 13.16, altitude 30 m and 325 m above sea level with a land area of about 69,435 square kilometers, operating on the West African time zone [23,24]. The study was conducted in six out of the eight secondary schools in Maiduguri Metropolitan Council (MMC), Borno State, Nigeria. Three out of the six secondary schools were single girls’ schools while the remaining three secondary schools were mixed with both boys and girls. The total population of the six secondary schools was 20,397, with a total of 14,672 girls.

### 2.2. Study Design, Population, and Sample Size

It was a school-based cross-sectional study conducted between 3 June and 31 July 2019 among adolescent girls aged 10 to 19 years’ old in six secondary schools in MMC, Borno state, Nigeria. Two proportion formula for sample size by Lemeshow and Lwanga was used to determine the sample size [25], with 0.05 margin of errors at 95% confidence interval (CI), the proportion of distribution based on religion among adolescent girls was *p*_1_ (0.47) and *p*_2_ (0.02) [26]. The calculated sample size was 424, considering the effect size of 1.3 and a 10% non-response rate, the total sample size was 612.

### 2.3. Sampling Procedure

A two-stage random sampling technique was used. First stage six out of the eight secondary schools in Maiduguri Metropolitan Council (MMC) were randomly selected using a random number generator, where all schools were given equal chances of participation. The second stage, the schools had six arms, junior secondary 1–3 (JSS 1–3) for the junior set and senior secondary 1–3 (SS 1–3) for the senior set. Two classes were randomly selected in each grade level/arm, and then finally, individuals from these classes were randomly selected. Six hundred and twelve adolescent girls were randomly selected; the number of adolescent girls from the six selected schools was determined using a proportionate sampling technique. Finally, adolescent girls aged 10 to 19 years were selected.

### 2.4. Data Collection

The instrument for data collection used was questionnaire through the respondent’s interview by enumerators using KoBo collect toolbox. A total of ten enumerators who were university graduate were trained for the data collection. Each interview lasted for 20 min per respondents, and the ten enumerators interviewed a total of 50 respondents per day. KoBo Toolbox used was an open and free data entry tool developed by Harvard Humanitarian initiative with support from various organizations like Brigham and Women’s Hospital, USAID [27]. The application can run on any device with android, be it phone or tablet. The application was freely downloaded, data collected was kept as cloud storage which was later transferred to SPSS for analysis. In this study, the global positioning system (GPS) coordinate of each school involved in the data collection was marked using the mobile phone.

### 2.5. Questionnaire

#### 2.5.1. Socio-Demographic Characteristics

The section is composed of fifteen questions to provide information on age, class, ethnicity, religion, place of residence, household size, household income, head of household, age of the father, father’s education, father’s occupation, age of mother, mother’s education, mother’s occupation, and family type.

#### 2.5.2. Knowledge on Malnutrition

This section consists of 28 questions on malnutrition. The options were “true”, “false”, or “don’t know”. Each correct response (true) was score one (1) point, and each wrong response (false) or “don’t know” was scored zero (0). The total knowledge score was 28. Knowledge items were categorized into scores ≥ 50% (14 to 28) were considered good knowledge, while <50% (14) were considered poor knowledge [28].

#### 2.5.3. Attitude on Malnutrition

This section consists of 17 statement on malnutrition. Attitude statement was rated on a five (5) point Likert scale (1 to 5), that ranged from Strongly disagree “1”, disagree “2”, don’t know “3”, agree “4”, and strongly agree “5”. Mean score for attitude statement was used to categorized attitude into two categories. Scores < than the mean score was considered a negative attitude while scores > the mean scores were considered positive attitude [29].

#### 2.5.4. Practice on Malnutrition

This section consists of two subsections, food consumption of 12 food groups and their meal frequency in seven days: Food consumption had a total score of 12 and meal frequency had a total score of 35. The total practice score was 47. The practice score was categorized into two groups. Scores below the median score were considered poor practice, while scores above the median score were considered as good practice [29].

#### 2.5.5. Information–Motivation–Behavioral Skills Construct (IMB)

This section consists of 16 statements from the IMB constructs. Information on malnutrition includes seven statements. The options for information were on a five (5) point Likert scale ranging from strongly disagree to strongly agree (1 to 5). Total information score ranges from 7 to 35. Information was categorized into two groups. Respondents who scored < the mean were considered poor information on malnutrition, while those above the mean were considered as having good information on malnutrition.

Motivation on malnutrition consisted of 4 statements. The options were on a five (5) point Likert scale ranging from strongly disagree to strongly agree (1 to 5). Total motivation score ranges from 4 to 20, and motivation was categorized into two-level. Respondents who scored < the median score were considered as having poor motivation on malnutrition, while above the median score were considered as having good motivation on malnutrition.

Behavioral skills on malnutrition consisted of 5 statements. The options were on a five (5) point Likert scale ranging from very hard to very easy. Very hard “1”, hard “2”, don’t know “3”, easy “4”, and very easy “5”. Total behavioral skill score ranges from 5 to 25. Behavioral skills were categorized into two groups. Scores < the median were considered poor behavioral skills on malnutrition, while those above the median score were considered as having good behavioral on malnutrition.

### 2.6. Data Analysis

Data collected were transferred from the cloud storage of KoBo collect toolbox to statistical package for social sciences (SPSS Inc., Chicago, IL, USA) software version 25 for analysis. Mean, and standard deviation was used to describe normally distributed continuous data, median and interquartile range was used to describe not normally distributed continuous data, frequencies and percentages were used to describe categorical data. Chi-square was used to test the association between each categorical independent variable with knowledge, attitude, and practice. Logistic regression was used to determine the predictors of knowledge, attitude, and practice of adolescent girls towards malnutrition. Factors with *p* < 0.25 in simple logistic regression were tested in multiple logistic regression. *p* < 0.05 was considered statistically significant. Y = α + β_1_X_1_ + β_2_X_2_ +…… β_N_X_N,_ where Y is the knowledge, attitude, practice; B is the slope of the line, X is the independent variable, and a is the intercept.

Spearman Rho correlation was used to analyze the association between the three IMB construct, further evaluation of IMB construct was done using structural equation model (SEM), where knowledge, attitude, and practice was explained by the three constructs (information, motivation, behavioral skills). The overall model fit was examined via standard indices (chi-square, comparative fit index (CFI), normed fit index (NFI), incremental fit index (IFI), Tucker Lewis index (TLI), parsimonious fit, and root mean square error of approximation (RMSEA)). Chi-square (*p* < 0.05), RMSEA < 0.08, CFI, NFI, IFI, and TLI value close to 0.9 or greater, parsimonious fit of <5.0 indicated good fit [21,30,31,32,33]. AMOS structural equation modelling software was used to assess the fit of the data to the IMB model. The current variables met the criterion for SEM normality with all variables within ≤2 skewness, and ≤7 kurtosis [34,35].

A pilot study was conducted in English among 60 adolescent girls in Lamisula senior secondary school and Yerwa practice school (UBE) located in the same school compound, in Maiduguri Metropolitan Council, Borno State, Nigeria. The Cronbach’s alpha coefficient for knowledge was 0.784, attitude 0.751. practice 0.801, information 0.761, motivation 0.844, and behavioral skills 0.878 were found to be acceptable greater than 0.7 [36]. Respondents were interviewed after two weeks to determine its reliability. All items for knowledge, attitude, practice, information, motivation, behavioral skills section had Cohen’s kappa score and intra-class correlation (ICC) of above 0.7.

Content validity of the questionnaire was assessed by six experts [37], five from the field of public health and a nutritionist. Twenty adolescent girls who were not part of the study were randomly selected to assess the questionnaire for face validity based on the order of question (good, average, or poor), language clarity (clear, average, confusing), and appropriateness (good, average, poor). The result shows that for knowledge section, 85% rated the order of the questions as good, 90% rated the language clarity as clear, and 75% rated the appropriateness for the construct as good. For the attitude section, 85% rated the order of the questions as good, 80% rated its language clarity as clear, while 85% rated its appropriateness as good. For the practice section, 85% rated the order of the questions as good, 80% rated the language clarity as clear, and 90% rated its appropriateness as good. For information section, 90% rated the order of questions as good, 85% rated language clarity as clear, while 90% rated its appropriateness as good. Motivation section, 90% rated the order of the question as good, 85% rated language clarity as clear, and 80% rated its appropriateness as good. Behavioral skills, 90% rated the order of question as good, 90% rated language clarity as clear, while 75% rated its appropriateness as good.

### 2.7. Ethical Approval and Consent

Ethical approval was obtained from the Jawatankuasa Etika Universiti Putra Malaysia UPM UPM/TNCPI/RMC/JKEUPM/1.4.18.2 (JKEUPM). The study was registered with the Pan African Clinical Trials Registry (PACTR201905528313816). The approval letter was obtained from the ministry of education Maiduguri, Borno State, Nigeria and an official letter of cooperation from the ministry of education Maiduguri was also written to each selected school. Signed informed consent was obtained from participants, and their parents/guardian in this study since the study population were adolescent girls younger than 18 years old.

## 3. Results

A total of 612 respondents were randomly selected based on the inclusion criteria of the study. Among the eligible respondents, 562 consented and were interviewed. The response rate was 92%.

### 3.1. Descriptive Analysis

#### 3.1.1. Socio-Demographic Characteristics of Respondents

Table 1 shows the sociodemographic characteristics of respondents consisting of 562 respondents. There were 108 (19.2%) respondents from Yerwa, 63 (11.2%) from GGSS, 86 (15.3%) from GGC, 72 (12.8%) from Shehu garbai, 149 (26.5%) from Bulabulin day, and 84 (14.9%) from Zajeri day. The age distribution of respondents was between 10 to 19 years, with a majority of 114 (20.3%) who are 16 years old. The median (IQR) age was 16 (3), majority of respondents were in the middle adolescent stage 280 (49.8%), 173 (30.8%) were in Senior secondary two (SS2), 173 (30.5%) were Kanuri, 447 (79.5%) were from the Islamic religion. Majority of respondents 309 (55.0%) have a household size of ≥9 members, 200 (35.6%) earned between 18,000 and 30,000, majority of their fathers 206 (37.2%) had tertiary education, while the majority of mothers had secondary educations 187 (33.6%). Trading/business was the main occupation of fathers and mothers 288 (52.7%) and 232 (42.4%), respectively. 510 (90.7%) had fathers as their head of households, and 281 (50.0%) were from monogamy family.

#### 3.1.2. Knowledge towards Reducing Malnutrition

Table 2 shows the distribution of correct and incorrect answers for knowledge items on malnutrition. Majority of respondents 367 (65.3%) know that carbohydrates are energy giving food, beans are sources of protein 389 (69.2%), proteins are body building food 376 (66.9%), butter, oil, nuts, meat, fish, milk contain fats 391 (69.6%), fruits and vegetables are sources of minerals and vitamins 373 (66.4%), eating different kinds of food can make us healthy 409 (72.8%), breakfast is the most important meal of the day 505 (89.9%).

According to this study, 509 (90.6%) are unaware that one of the causes of malnutrition is not eating enough food, 521 (92.7%) do not know that meat is a source of protein, 545 (97%) are not aware that lack of iron in the diet can cause anemia, 533 (94.5%) did not agree that iodine deficiency can be caused by eating or preparing foods with salt that is not iodized, and 511 (90.9%) disagree that calcium is a mineral that makes the bones strong and healthy. The median and (IQR) for knowledge score was 9 (6). Majority of respondents 459 (81.7%) had poor knowledge of malnutrition.

#### 3.1.3. Attitude towards Reducing Malnutrition

Table 3 describes the frequency and percentage of the respondents’ attitude scores towards malnutrition for each item. The mean and standard deviation for the attitude of respondents on malnutrition was 53 ± 7.6. Majority of respondents 322 (57.3%) had a poor attitude on malnutrition.

#### 3.1.4. Practice towards Reducing Malnutrition

The medium (IQR) for food consumption was 4 (2), medium (IQR) for meal frequency was 25 (9). Median IQR for practice was 29.5 (9). 278 (49.5%) of respondents had a poor practice towards reducing malnutrition as presented in Table 4.

#### 3.1.5. Information, Motivation, and Behavioral Skills of Respondents towards Reducing Malnutrition

Table 5 shows that mean ± SD for information was 21 ± 4.0. A higher number of respondents 387 (68.9%) had poor information on malnutrition, while 175 (31.1%) had good information on malnutrition. The median and IQR of Motivation was 16 (3), majority of respondents 436 (77.6%) had a poor level of motivation on malnutrition, while 126 (22.4%) had good motivation on malnutrition. The median and IQR of behavioral skills was 11 (5). Majority of respondents 291 (51.8%) had poor behavioral skills on malnutrition, while 271 (48.2%) had good behavioral skills on malnutrition.

#### 3.1.6. Sources of Information

**Figure 3:**
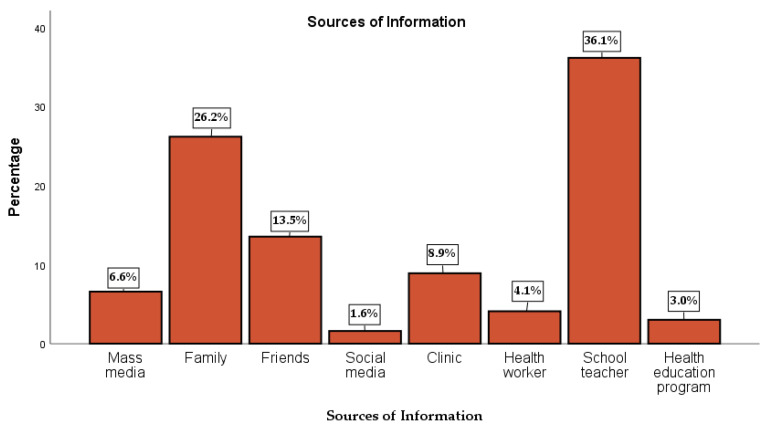
Respondents Regular Sources of Nutritional Information.

The sources of information of majority of respondents were school teacher 36.1%, media 6.6%, family 26.2%, friends 13.5%, social media 1.6% (WhatsApp and Facebook), clinic 8.9%, health worker 4.1%, and health education program 3.0% as shown in Figure 3.

### 3.2. Association between Sociodemographic Characteristics, Information, Motivation, and Behavioral Skills with Knowledge, Attitude, and Practice of Adolescent Girls towards Reducing Malnutrition

The bivariate analysis shows that schools (*p* < 0.001) were significantly associated with knowledge towards reducing malnutrition. Information (*p* < 0.001), motivation (*p* < 0.001) and behavioral skills on malnutrition (*p* = 0.024) were significantly associated with the attitude towards reducing malnutrition. Schools (*p* < 0.001), monthly income (*p* = 0.044), education of father (*p* = 0.009), head of household (*p* = 0.035), information (*p* = 0.035), and behavioral skills on malnutrition (*p* < 0.001) were significantly associated with practice towards reducing malnutrition.

#### 3.2.1. Predictors of Knowledge, Attitude, and Practices towards Reducing Malnutrition

School, age of adolescent, class, ethnicity, religion, place of residence, monthly income, and education of father with *p* < 0.25 were included in multivariable logistic regression for predicting knowledge towards reducing malnutrition. Findings of multivariable logistic regression analysis in Table 5 indicated that school was a predictor of knowledge towards reducing malnutrition. Respondents in Government Girls Secondary School Maiduguri (GGSS) and Shehu Garbai Secondary School were less likely to have good knowledge towards reducing malnutrition as compared to respondents in Yerwa (AOR = 0.303, 95% CI: 0.109–0.842, *p* = 0.022; AOR = 0.150, 95% CI: 0.043–0.521, *p* = 0.003), respectively.

School, age of adolescent, class, ethnicity, religion, monthly income, head of household, education of father, occupation of father, age of mother, education of mother, occupation of mother, information, motivation, and behavioral skills with *p* < 0.25 were included in multivariable logistic regression for predicting attitude towards reducing malnutrition. Religion, information, and motivation on malnutrition were significant predictors of attitude towards reducing malnutrition. Respondents in Islamic religion were less likely to have good attitude towards reducing malnutrition as compared to Christians (AOR = 0.513, 95% CI: 0.384–0.932, *p* = 0.023). Respondents with good information on malnutrition were more likely to have a good attitude towards reducing malnutrition as compared to those with poor information on malnutrition (AOR = 2.584, 95% CI: 1.736–3.846, *p* < 0.001). Respondents with good motivation on malnutrition were more likely to have a good attitude towards reducing malnutrition as compared to those with poor motivation on malnutrition (AOR = 3.334, 95% CI: 2.144–5.186, *p* < 0.001)

School, ethnicity, religion, place of residence, monthly income, head of household, age of father, education of father, age of mother, education of mother, occupation of mother, family type, information, motivation, and behavioral skill with *p* < 0.25 were included in multivariable logistic regression for predicting practice towards reducing malnutrition. School, education of father, information and behavioral skills on malnutrition were significant predictors of practice towards reducing malnutrition. Respondents from GGSS, Government Girls College (GGC), Shehu garbai secondary school, bulabulin, zajeri day secondary were more likely to have good practice towards reducing malnutrition as compared to those from Yerwa (AOR = 3.866, 95% CI: 1.914–7.809, *p* < 0.001; AOR = 4.545, 95% CI: 2.381–8.674, *p* < 0.001; AOR = 8.319, 95% CI: 4.006–17.279, *p* < 0.001; AOR = 1.862, 95% CI: 1.063–3.263, *p* = 0.030; AOR = 1.908, 95% CI: 1.003–3.628, *p* = 0.049). Respondents whose fathers had informal education were less likely to have good practice towards reducing malnutrition as compared to those whose fathers had no education (AOR = 0.305, 95% CI: 0.122–0.600, *p* = 0.001). Respondents with good information on malnutrition were more likely to have good practice towards reducing malnutrition as compared to those with information AOR = 1.595, 95% CI: 1.058–2.404, *p* = 0.026). Respondents with good behavioral skills on malnutrition were more likely to have good practice towards reducing malnutrition as compared to those with poor behavioral skills AOR = 1.572, 95% CI: 1.077–2.296, *p* = 0.019) as presented in Table 6.

#### 3.2.2. Information, Motivation, Behavioral skills (IMB) Model and Knowledge, Attitude, Practice

IMB model shows the association between its construct. Information was statistically associated with behavioral skills (*p* = 0.018), motivation was statistically associated with behavioral skills (*p* < 0.001), while behavioral skills were statistically associated with information (*p* = 0.018) and motivation (*p* < 0.001).

IMB also shows statistically significant association with knowledge, attitude, and practice towards reducing malnutrition. Information had a statistically significant association with behavioral skills (*p* = 0.018), knowledge (*p* = 0.002), and attitude (*p* < 0.001). Motivation had a statistically significant association with behavioral skills (*p* < 0.001), knowledge (*p* < 0.001), attitude (*p* < 0.001) and practice (*p* = 0.001). More so, behavioral skills reveal statistically significant association with information (*p* = 0.018), motivation (*p* < 0.001), attitude (*p* < 0.001) and practice (*p* < 0.001).

Structural equation model based on the IMB construct and knowledge, attitude, and practice showed statistical association in all the paths. Information and knowledge (*p* = 0.002), motivation and knowledge (*p* < 0.001), motivation and practice (*p* = 0.016), behavioral skill and practice (*p* < 0.001), information and attitude (*p* < 0.001), motivation and attitude (*p* = 0.004) lastly, behavioral skills and attitude (*p* = 0.014).

**Figure 4:**
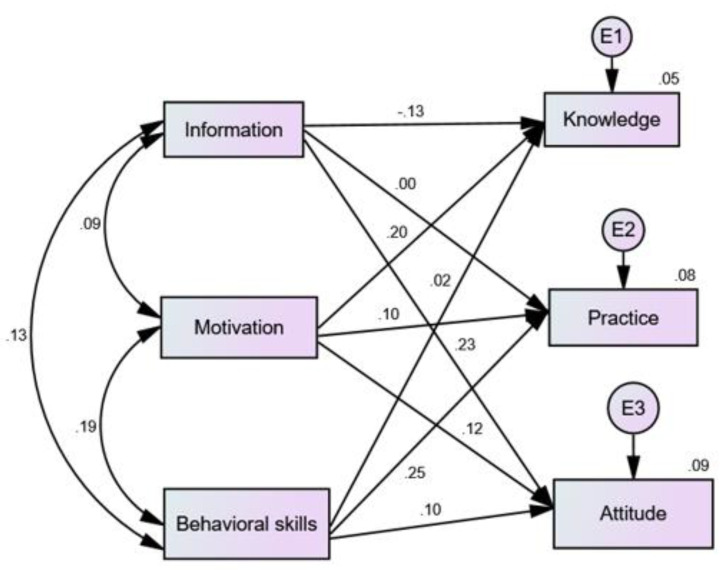
Structural equation model. The measure used in the model: Information; Motivation; Behavioral skills; Knowledge; Attitude; Practice.

The structural equation model supported the theoretical relationship between IMB and Knowledge, attitude, and practice, as shown in Figure 4. The path between (a) motivation and knowledge, (b) motivation and practice, (c) behavioral skill and practice, (d) information and attitude, (e) motivation and attitude and (f) behavioral skill and attitude were all positive and significant. The path between (a) information and knowledge were negative and significant. Standard indices in this study showed that the model is fit were X^2^ = 10.121, DF = 3, *p* = 0.018, CFI = 0.956, IFI = 0.959, NFI = 0.942, TLI = 0.778, parsimony adjusted measures = 0.191, RMSEA = 0.065.

## 4. Discussion

This study aims at assessing the knowledge, attitude, and practice of adolescent girls towards malnutrition. Nutrition-related problems early in life have an impact on the well-being and quality of life of adolescents. They can have far-reaching implications for the adolescents now, when they become adult and on their unborn children, thereby affecting the country’s economic growth, both in terms of lost productivity and increased burden of disease. The study demonstrated that majority of respondents were in their middle adolescent stage 280 (49.8%), majority 309 (55.0%) had household size ≥9 members, majority of fathers 206 (37.2%) had tertiary education while the majority of mothers 187 (33.6%) had secondary school education, trading/business was the occupation of the majority of fathers 288 (52.7%) and mothers 232 (42.4%), while 510 (90.7% had their fathers as head of household. The study further reveals that majority of respondents (459, 81.7%; 322, 57.3%) had poor knowledge and attitude towards reducing malnutrition respectively, 278 (49.5%) had poor practice towards reducing malnutrition. A higher number of respondents (387, 68.9%; 436, 77.6%; 291, 51.8%) had poor information, motivation, and behavioral skill towards reducing malnutrition. The majority of the respondent’s source of information was the schoolteacher. The school was a predictor of knowledge towards reducing malnutrition. Religion, information, and motivation were predictors of attitude towards reducing malnutrition. School, education of father, information, and behavioral skills were predictors of practice towards reducing malnutrition.

The majority of respondents in this study were in their middle adolescent stage 280 (49.8%) this is in line with a study conducted in northwest Ethiopia, 2017, Ibadan Nigeria 2014, and Southwest Nigeria 2017, who reported majority 402 (52.3%), 68 (73.1%), and 180 (71.7%) respectively of adolescent girls were in middle adolescent stage [14,15,38]. The majority of respondents in this study have a family size of greater than 9 which is contrary to the study conducted in Ibadan, Nigeria, and West Bengal, India who reported majority ≤4 family members 62 (66.7%) and 5–7 family members 324 (57.86%) [14,39]. The findings in this study may be associated with the polygamous nature of the Northern region of Nigeria. Majority of fathers in this study had tertiary education 206 (37.2%), this is in line with a study conducted in Jimma Town, Ethiopia 2017, and Ibadan 2014 who reported post-secondary education 171 (37.6%) and 160 (64.5%) as the majority of father’s education respectively. Majority of mothers in this study 187 (33.6%) had secondary school education which is contrary to the study conducted in Ibadan Nigeria, 2014, who reported the majority of mothers with post-secondary education. These may be associated with a low level of women education in the northern part of Nigeria as compared to the southern region [40].

This study revealed that the respondents have poor knowledge towards reducing malnutrition 451 (80.2%) which is contrary to studies elsewhere in Southwest Ethiopia, Nairobi city, Kenya, Palestine, and Darab city, Fars province, Iran where 201 (44.2%), 32.7%, 52.4%, and 38.5% of the respondents had poor knowledge, respectively [26,41,42]. Findings from studies conducted in Sharjah, United Arab Emirates reveals that 86% of adolescents had poor knowledge of nutrition [43]. Results in this study reported that breakfast is the most important meal of the day 89.9%, this is in line with studies conducted in China who reported 68.8% and South Africa who reported 87.6% [44,45]. Of adolescent girls in the study, 92.7% were not aware that meat is a source of protein which is contrary to study conducted in Tirupati, India which reported 67% of the girls were aware that meat is a source of protein [8]. Findings from other studies could be because most of the respondents were familiar with the negative impact of malnutrition-related consequences, deficiencies of different types of micronutrients like iron, iodine, vitamin A, and their varying effects. Nutrition education from the various non-governmental organization in Ethiopia has positively influence adolescent girls towards healthy living. Also, findings in Ghana showed that adolescents girls have more knowledge of dietary-related consequences [26,46]. The study hypothesized that adolescents were not aware of the definition, causes, forms, consequences of malnutrition, furthermore, adolescent girls in this study were unaware of micronutrient and its implications.

Adolescent girls in this study have poor knowledge of definition, forms, causes, consequences, and preventive measures of malnutrition, micronutrient deficiencies like iron, iodine, vitamin A, and calcium. Poor knowledge towards malnutrition in this study may be influenced by the type of schools the adolescent girls attend since the study was carried out in government secondary schools where the majority of respondents were from the middle and lower-class families. Also, poor knowledge may be contributed to by the lack of exposure of the girls to varieties of nutritional health education in the schools as all attention in the northeast of Nigeria has been geared towards under-five children and pregnant and lactating mothers, overlooking the adolescents who are the future mothers.

Majority of respondents 322 (57.3%) in this study had a poor attitude towards reducing malnutrition which is in line with the study conducted in Sulawesi, Indonesia where 55.5% of respondents had a negative attitude towards reducing malnutrition [47]. The poor attitude of adolescent girls towards reducing malnutrition may play an essential role in the quality and quantity of food they eat. This may affect their growth, development, and pose a serious health risk because the majority of them do not believe that they can be malnourished and deficient in iron and vitamin A. However, they see malnutrition as a condition that affects only children under the age of five years. More so, the finding in this study revealed that less than 50% of adolescent girls have poor practice towards malnutrition which is in line with a previous study in Indonesia where 46.5% of respondents had poor malnutrition practice [47].

This study further revealed an association between schools and knowledge of adolescent girls towards reducing malnutrition. This shows that being in school increases the chance of having good knowledge. In this study, information, motivation, and behavioral skill (IMB) on malnutrition were associated with the attitude towards reducing malnutrition. These imply that the use of IMB theory in studies might help adolescent girls in holding holistic perception of their behavior. The purpose of the IMB model recommends that well informed, motivated individuals who have the required behavioral skills among adolescents were found to be more likely to maintain healthy behavior resulting to positive health outcome [48,49]. There was a statistically significant association between practice towards reducing malnutrition with school, monthly household income, education of fathers, head of household, information, and behavioral skills. This is in line with a previous study in Swaziland and Jimma town, Ethiopia in 2014 who reported an association between practice, monthly income, educational level, and schools [26,50,51].

Multivariate logistic regression analysis in this study hypothesized that school was a predictor of knowledge towards reducing malnutrition. Religion, information, and motivation were predictors of attitude towards reducing malnutrition. School, education of father, information, and behavioral skills were predictors of practice towards reducing malnutrition. Our results indicate that adolescent girls whose fathers had no education were less likely to have good practice towards reducing malnutrition, which could be associated with inaccessibility to nutrition-related information on the consequences of malnutrition.

The study further examines whether the IMB model can be applied to explain the level of knowledge, attitude, and practices towards reducing malnutrition among adolescent girls. The study reveals a statistically significant association between information, motivation, behavioral skills with knowledge, attitude, and practice. This indicates that adequate and comprehensive information, motivation and behavioral skill would improve knowledge, attitude, and practice of adolescent girls. The authors hypothesized that a higher level of information, motivation and behavioral skills would be significantly associated with improved knowledge, attitude, and practice.

The findings further reveal that information on its own is not sufficient to bring about the required behavioral change. Still, when combined with the appropriate motivation, it helps in equipping the individual with the necessary skills and competence to have the desired behavior for prevention and promotion purposes. Subsequently, this will lead to a good behavioral outcome. This is similar to studies conducted on adherence to medicine which reveals that information was not significant with medication adherence [52]. Studies from systematic review show the potential strength of the IMB model as a theoretical frame to developing study instrument [53]. The study further reiterated the usefulness of IMB in reducing risky behavior and improving positive health outcome among adolescents and youth [54].

The adolescent stage is a unique point of intervention in their life cycle, that will not only address the issue of malnutrition in this phase of their life but will also tackle malnutrition beyond the 1000 day among future offspring’s. The finding from this study reveals a great lack of knowledge, attitude, and practice towards reducing malnutrition. Without active intervention, adolescent girls are at risk of malnutrition, and other associated health problem. Therefore, the findings from this study can play an essential role in designing and implementing appropriate interventions to improve the health outcome among adolescent girls. To the best of our knowledge, this is the first time the IMB model was evaluated among adolescents towards reducing malnutrition in Nigeria. Importantly, the model shows the association between the IMB constructs, which are essential to improving knowledge, attitude, and practice among adolescent girls. Adequate nutritional needs remain throughout life, enhancing the quality of life.

### Limitations

The study population were adolescent girls from six secondary schools. This does not represent adolescent girls attending private schools, the school dropped out and those not attending any school at all in Maiduguri metropolitan council, who may be at risk of malnutrition due to inadequate knowledge, attitude, and practice towards reducing malnutrition. The use of self-reported questionnaire may also be seen as a limitation.

## 5. Conclusions

Ignorance about malnutrition, macro and micronutrient prevailed among adolescent girls. Access to knowledge, attitude, and practice towards malnutrition was poor for adolescent girls. Increasing knowledge on malnutrition and its related consequences may improve the attitude and practice of adolescent girls towards reducing malnutrition and a healthy lifestyle. Findings from this study have identified the gaps in knowledge, attitude, and practice towards reducing malnutrition among adolescent girls. The study suggests that there is a need to provide a comprehensive and sound health education intervention on malnutrition, for the present and future health benefits of adolescent girls. When adolescent girls become healthy at this phase, they will grow to be healthy adults and in turn, have a healthy and productive offspring. Future studies to inform the effective development and implementation of a theory-based intervention for adolescent girls enrolled in schools and out of school will go a long way, leading to a healthy life as adolescent girls, adults, and also to healthier offspring that will translate into a healthier community.

## Figures and Tables

**Table 1:** Sociodemographic characteristics of respondents (*n* = 562).

Variables	Classification	Frequency (*n* = 562)	Percentage (%)
Schools ^a^	Yerwa	108	19.2
	GGSS	63	11.2
	GGC	86	15.3
	Shehu Garbai	72	12.8
	Bulabulin day	149	26.5
	Zajeri day	84	14.9
Age of adolescent girls (years)	Early adolescents	100	17.8
	Middle adolescents	280	49.8
	Late adolescents	182	32.4
Class ^b^	JSS1	132	23.5
	JSS2	110	19.6
	SS1	147	26.2
	SS2	173	30.8
Ethnicity	Bura	48	8.5
	Kanuri	173	30.5
	Hausa	58	10.3
	Marghi	52	9.3
	Shuwa	28	5.0
	Fulani	52	9.3
	Chibok	19	3.4
	Gwoza	87	15.5
	Other ethnic group ^c^	45	8.0
Religion	Christianity	115	20.5
	Islam	447	79.5
Place of residence	Urban	448	86.8
	Rural	74	13.2
Household size	≤5 members	46	8.2
	6–8 members	207	36.8
	≥9 members	309	55.0
Monthly income	>18,000	163	29.0
	18,000–30,000	200	35.6
	31,000–50,000	139	24.7
	≥51,000	60	10.7
Head of household	Father	510	90.7
	Mother	31	5.5
	Relations	21	3.7
Age of father (years)	≤34		
	35–44	52	9.6
	≥45	488	90.4
Education of father	No education	57	10.3
	Informal education	82	14.8
	Primary education	16	2.9
	Secondary education	193	34.8
	Tertiary education	206	37.2
Occupation of fathers	Civil service	196	35.9
	Trading/business	288	52.7
	Farming	41	7.5
	Other occupation ^d^	21	3.8
Age of mother (years)	≤34	100	18.1
	35–44	280	50.6
	≥45	173	31.3
Education of mothers	No education	101	18.1
	Informal education	123	22.1
	Primary education	58	10.4
	Secondary education	187	33.6
	Tertiary education	88	15.8
Occupation of mothers	Civil service	86	15.7
	Trading/business	232	42.4
	Farming	27	4.9
	House wives	202	36.9
Family type	Monogamy	281	50.0
	Polygamy	239	42.5
	single parenting	42	7.5

^a^ Government girls’ secondary school Yerwa, Government girls secondary school Maiduguri, Government girl’s college Maiduguri, Shehu garbai day secondary school, Bulabulin day secondary school, Zajeri day secondary school. ^b^ Junior secondary school (JSS), Senior secondary school (SS) ^c^ Karekare, Kilba, Minchika, Manga, Tambai, Yoruba, Mandara, Basaye, Angas, Terawa, Kanakuru, Nupe. ^d^ Malami.

**Table 2:** The distribution of correct and incorrect responses to knowledge questions on malnutrition.

Knowledge Item on Malnutrition	Correct (%)	Incorrect (%)
Malnutrition refers to deficiencies in an individual nutritional intake	199 (35.4)	363 (64.6)
Undernutrition is a form of malnutrition	50 (8.9)	512 (91.1)
One of the causes of malnutrition is not eating enough food	212 (37.7)	350 (62.3)
Slow growth in adolescence is a sign of malnutrition	53 (9.4)	509 (90.6)
Muscle wasting is a sign of malnutrition	108 (19.2)	454 (80.8)
Stunted growth can be a result of malnutrition	41 (7.3)	521 (92.7)
One of the ways through malnutrition can be prevented by eating frequently.	203 (36.1)	359 (63.9)
Carbohydrates, protein, fats are an essential nutrient that the body needs in large amount for growth	137 (24.4)	425 (75.6)
Cereals are a good source of carbohydrate	205 (36.5)	357 (63.5)
Carbohydrates are energy giving food	367 (65.3)	195 (34.7)
Beans are sources of protein	389 (69.2)	173 (30.8)
Proteins are body building food	376 (66.9)	186 (33.1)
The body uses fat as a source of energy	227 (40.4)	335 (59.6)
Butter, oil, nuts, meat, fish, milk contain fats	391 (69.6)	171 (30.4)
Vitamins and minerals are essential nutrients that the body needs in small amounts to function properly	132 (23.5)	430 (76.5)
Fruits and vegetables are sources of minerals and vitamins	373 (66.4)	189 (33.6)
Anaemia can be caused by insufficient iron in the body.	17 (3.0)	545 (97.0)
Lack of iron in the diet can cause anaemia	17 (3.0)	545 (97.0)
Slow physical growth can be caused by insufficient iron in the body	21 (3.7)	541 (96.3)
Meat is a source of iron	41 (7.3)	521 (92.7)
Pumpkin, (kabewa), carrots, green vegetables are good sources of vitamin A	141 (25.1)	421 (74.9)
Night blindness is not a sign of insufficient vitamin A in the body *	24 (4.3)	538 (95.7)
Iodine deficiency can be caused by eating or preparing foods with salt that is not iodized	29 (5.2)	533 (94.5)
Goitre is a sign of a lack of iodine in the body	31 (5.5	531 (94.5)
Calcium is a mineral that makes the bones strong and healthy	51 (9.1)	511 (90.9)
Milk or green vegetables or beans are good sources of calcium	160 (28.5)	402 (71.5)
Eating different kinds of food can make us healthy	409 (72.8)	153 (27.2)
Breakfast is the most important meal of the day	505 (89.9)	57 (10.1)
Poor knowledge towards malnutrition	451 (80.2%)	
Good knowledge towards malnutrition	111 (19.8%)	

* Negative statements.

**Table 3:** The distribution of responses to attitude statements on malnutrition.

Attitude Item on Malnutrition	Strongly Disagree *n* (%)	Disagree *n* (%)	Neutral *n* (%)	Agree *n* (%)	Strongly Agree *n* (%)
I think I may be malnourished	154 (27.4)	242 (43.1)	105 (18.7)	36 (6.4)	25 (4.4)
I think poverty is one of the causes of malnutrition.	41 (7.3)	71 (12.6)	78 (13.9)	301 (53.6)	71 (12.6)
Malnutrition is a serious problem in adolescents	18 (3.2)	75 (13.3)	158 (28.1)	240 (42.7)	71 (12.6)
I think noodles have more nutrients than food cereals *	48 (8.5)	269 (47.9)	109 (19.4)	101 (18.0)	35 (6.2)
I think adolescent girls do not need food containing fat at this stage *	27 (4.8)	202 (35.9)	177 (31.5)	125 (22.2)	31 (5.5)
I think I can get energy from protein only *	23 (4.1)	128 (22.8)	132 (23.5)	233 (41.5)	46 (8.2)
I think I may be iron deficient	27 (4.8)	157 (27.9)	282 (50.2)	61 (10.9)	35 (6.2)
I think I may not have sufficient vitamin A in my body	34 (6.0)	183 (32.6)	234 (41.6)	79 (14.1)	32 (5.7)
I think processed juices (e.g., chivita, five alive, faro) are more nutritious than fresh fruits (e.g., oranges, watermelon, pineapple, mango) *	67 (11.9)	294 (52.3)	94 (16.7)	71 (12.6)	36 (6.4)
I think it is good to prepare meal with iodized salt	17 (3.0)	93 (16.5)	174 (31.0)	240 (42.7)	38 (6.8)
I think it is only older people that suffer from calcium deficiency *	52 (9.3)	196 (34.9)	198 (35.2)	87(15.5)	29 (5.2)
I think when I take breakfast, I perform better in school	26 (4.6)	77 (13.7)	84 (14.9)	233 (41.5)	142 (25.3)
I think eating three times a day makes me perform better in school	13 (2.3)	18 (3.2)	56 (10.0)	282 (50.2)	193 (34.3)
I think the taste of food is more important than its nutritional quality *	28 (5.0)	147 (26.2)	121 (21.5)	218 (38.8)	48 (8.5)
I think expensive foods are the most healthy foods *	21 (3.7)	115 (20.5)	86 (15.3)	267 (47.5)	73 (13.0)
I think I can be healthy even if I don’t eat varieties of food *	32 (5.7)	137 (24.4)	122 (21.7)	228 (40.6)	43 (7.7)
I take less nutritious food to have a slim shape *	42 (7.5)	231 (41.1)	164 (29.2)	96 (17.1)	29 (5.2)
Poor attitude towards malnutrition	322 (57.3%)				
Good attitude towards malnutrition	240 (42.7%)				

* Negative statement.

**Table 4:** Summary of practice towards reducing malnutrition.

Variable	Medium (IQR)/*n* (%)
Food consumption score	4(2)
Meal frequency score	25.00(9)
Total (practice score)	29.50 (9)
Poor practice (scores <29.5 below Median)	278 (49.5%)
Good practice (scores ≥29.5 above Median)	284 (50.5%)

**Table 5:** Information of on malnutrition.

**Information Item**	**Strongly Disagree** ***n* (%)**	**Disagree** ***n* (%)**	**Neutral** ***n* (%)**	**Agree** ***n* (%)**	**Strongly Agree** ***n* (%)**
The best way to prevent malnutrition is to eat different kinds of food	18 (3.2)	87 (15.5)	154 (27.4)	182 (32.4)	121 (21.5)
What I eat makes me malnourished	33 (5.9)	241 (42.9)	172 (30.6)	86 (15.3)	30 (5.3)
There is no food that can help prevent malnutrition *	37 (6.6)	226 (40.2)	163 (29.0)	108 (19.2)	28 (5.0)
If you are malnourished, there is nothing you can do about it *	56 (10.0)	219 (39.0)	168 (29.9)	97 (17.3)	22 (3.9)
Malnutrition makes people to be tired always	18 (3.2)	88 (15.7)	234 (41.6)	182 (32.4)	40 (7.1)
I do not care about eating the right foods for malnutrition *	31 (5.5)	168 (29.9)	209 (37.2)	129 (23.0)	25 (4.4)
With everything else that is going on in my life, I don’t care about finding foods that can prevent me from being malnourished *	32 (5.7)	181 (32.2)	202 (35.9)	125 (22.2)	22 (3.9)
**Motivation**	**Strongly Disagre** ***n* (%)**	**Disagree** ***n* (%)**	**Neutral** ***n* (%)**	**Agree** ***n* (%)**	**Strongly Agree** ***n* (%)**
I think I like eating different kinds of foods	11 (2.0)	45 (8.0)	59 (10.5)	324 (57.7)	123 (21.9)
I think the people who are important to me (family and friends) like eating different kinds of food	8 (1.4)	52 (9.3)	100 (17.8)	324 (57.7)	78 (13.9)
I think the people who are important to me (family and friends) always encourage me to eat different kinds of food	10 (1.8)	46 (8.2)	101 (18.0)	345 (61.4)	60 (10.7)
I think the people who are important to me (friends and family) always encourage me to buy nutritious food	9 (1.6)	47 (8.4)	108 (19.2)	337 (60.0)	61 (10.9)
**Behavioral Skills**	**Very Hard** ***n* (%)**	**Hard** ***n* (%)**	**Neutral** ***n* (%)**	**Easy** ***n* (%)**	**Very Easy** ***n* (%)**
How hard or easy is it for you to buy nutritious food?	47 (8.4)	303 (53.9)	69 (12.3)	129 (23)	14 (2.5)
How hard or easy is it for you to buy nutritious food within your current budget for food?	58(10.3)	307 (54.6)	73 (13.0)	112 (19.9)	12 (2.1)
How hard or easy is it for you to buy and store fish, rice, meat, or fruits at home?	81 (14.4)	298 (53.0)	72 (12.8)	98 (17.4)	13 (2.3)
How hard or easy is it for you to cook nutritious food?	48 (8.5)	264 (47.0)	101 (18.0)	126 (22.4)	23 (4.1)
How hard or easy is it for you to cook nutritious food for your family members	51 (9.1)	276 (49.1)	93 (16.5)	125 (22.2)	17 (3.0)

* Negative statements.

**Table 6:** Multivariable logistic regression model predicting the knowledge, attitude, and practice of adolescent girls towards reducing malnutrition.

Variable	Knowledge				Attitude				Practice			
	B	Adjusted Odd Ratio	95% CI	*p*-Value	B	Adjusted Odd Ratio	95% CI	*p*-Value	B	Adjusted Odd Ratio	95% CI	*p*-Value
Name of school												
Yerwa	Ref								Ref			
GGSS	−1.193	0.303	0.109–0.842	0.022 *	-	-	-	-	1.352	3.866	1.914–7.809	<0.001 **
GGC	0.635	1.886	0.999–3.562	0.050	-	-	-	-	1.514	4.545	2.381–8.674	<0.001 **
Shehu Garbai	−1.895	0.150	0.043–0.521	0.003 *	-	-	-	-	2.119	8.319	4.006–17.279	<0.001 **
Bulabulin Day	−0.488	0.614	0.323–1.166	0.136	-	-	-	-	0.622	1.862	1.063–3.263	0.030 *
Zajeri	0.474	1.606	1.606–3.076	0.153	-	-	-	-	0.646	1.908	1.003–3.628	0.049 *
Religion	-	-	-	-								
Christianity	-	-	-	-	Ref				-	-	-	-
Islam	-	-	-	-	−0.461	0.631	0.400–0.994	0.047 *	-	-	-	-
Education of father												
No education	-	-	-	-	-	-	-	-	Ref			
Informal education	-	-	-	-	-	-	-	-	−1.305	0.271	0.122–0.600	0.001 *
Primary education	-	-	-	-	-	-	-	-	−1.117	0.327	0.092–1.158	0.083
Secondary education	-	-	-	-	-	-	-	-	−0.578	0.561	0.283–1.12	0.098
Tertiary education	-	-	-	-	-	-	-	-	−0.467	0.652	0.332–1.282	0.215
Information												
Poor level of information	-	-	-	-	Ref				Ref			
Good level of information	-	-	-	-	0.949	2.584	1.736–3.846	<0.001 **	0.467	1.595	1.058–2.404	0.026 *
Motivation												
Poor level of motivation	-	-	-	-	Ref				-	-	-	-
Good level of motivation	-	-	-	-	1.204	3.334	2.144–5.186	<0.001 **	-	-	-	-
Behavioral skills												
Poor behavioral skill level	-	-	-	-	-	-	-	-	Ref			
Good behavioral skill level	-	-	-	-	-	-	-	-	0.452	1.572	1.077–2.296	0.019 *
Intercept	−1.241	0.289			−0.490	0.613			−0.642	0.526		

* Significant at *p* < 0.05, ** Significant at *p* < 0.001, CI = Confidence Interval of adjusted odd ratio, Ref = Reference category, Method = Backward stepwise method, Yerwa = Government girls’ secondary school Yerwa, GGSS = Government girls secondary school Maiduguri, GGC = Government girl’s college Maiduguri, Shehu garbai day secondary school, Bulabulin day secondary school, Zajeri day secondary school.
